# Statistical inference and effect measures in abstracts of major HIV and AIDS journals, 1987–2022: A systematic review

**DOI:** 10.1016/j.gloepi.2025.100213

**Published:** 2025-07-25

**Authors:** Andreas Stang, Henning Schäfer, Ahmad Idrissi-Yaghir, Christoph M. Friedrich, Matthew P. Fox

**Affiliations:** a Institute for Medical Informatics, Biometry, and Epidemiology (IMIBE), University Hospital Essen, Hufelandstr. 55, 45147 Essen, Germany; b School of Public Health, Department of Epidemiology, Boston University, 715 Albany Street, Boston, MA 02118, USA; c Departments of Epidemiology and Global Health, Boston University School of Public Health, Boston University, 715 Albany Street, Boston, MA 02118, USA; d Department of Computer Science, University of Applied Sciences and Arts Dortmund (FHDO), Emil-Figge-Str. 42, 44227 Dortmund, Germany; e Institute for Transfusion Medicine, University Hospital Essen, Hufelandstr. 55, 45147 Essen, Germany; f Institute for Artificial Intelligence in Medicine (IKIM), University Hospital Essen, Giradetstr. 2, 45131 Essen, Germany

**Keywords:** HIV, Acquired immunodeficiency syndrome, Confidence intervals, Statistics, Statistics and numerical data

## Abstract

**Objectives:**

With the emergence of HIV/AIDS journals, the development of the reporting of statistical inference and effect measures in published abstracts can be examined from the beginning in a new field. The aim of this study was to describe time trends of statistical inference and effect measure reporting of major HIV/AIDS journals

**Methods:**

We included 10 major HIV/AIDS journals and analyzed all available PubMed entries for the period 1987 through 2022. We applied rule-based text mining and machine learning methodology to detect the presence of confidence intervals, numerical *p*-values or comparisons of p-values with thresholds, language describing statistical significance, and effect measures for dichotomous outcomes

**Results:**

Among 41,730 PubMed entries from the major HIV/AIDS journals, 31,665 contained an abstract. In the early years, most abstracts reporting statistical inference contained only significance terminology without confidence intervals and *p*-values. From 1988 to 2005, each year 30 % of all abstracts contained p-values without confidence intervals. Thereafter, this reporting style continued to decline. The reporting of confidence intervals increased steadily from 1988 (11 %) to 2022 (56 %). Of the 17 % of abstracts in 2017–2022 that included any effect measure, half reported odds ratios (51 %), followed by hazard ratios (28 %) and risk ratios (16 %). Difference measures and number needed to treat or harm were very uncommon

**Conclusions:**

Within the HIV/AIDS literature, there has been widespread use of confidence intervals. Most of the journals that we reviewed had a decrease in reporting only statistical significance without confidence intervals over time

## Highlights

• Initially, statistical reporting contained only significance terminology.
• Reporting of confidence intervals increased from 1988 (11 %) to 2022 (56 %).
• Most frequently reported effect measures included odds, hazard and risk ratios.
• Difference measures and number needed to treat or harm were very uncommon.

## Introduction

There have long been calls in the field of epidemiology to move away from null hypothesis significance testing (NHST) and, less so, use of *p*-values, for evaluating random error. Yet NHST continues to be common in the fields of epidemiology and medical research, even if it may be more subtle than it used to be [[1], [2], [3], [4], [5]]. The critique focuses on a number of problems with the NHST approach, including the fact that p-values do not have a clear probability interpretation in observational studies with residual bias [6], problems with dichotomization of a continuous measures of evidence into significant or not, the fact that significance has been taken to imply meaningfulness at the expense of clinical significance, the obsessive focus on the null hypotheses at the expense of all other hypotheses the data might be consistent with that NHST encourages [7], the tendency of NHST to lead to mostly publishing significant results (publication bias) of which many are false positive results [8], the lack of focus on precision and estimation [9], and numerous other arguments [10].

This push to make researchers aware of the limitations of *p*-values and NHST has come with a push towards the use of confidence intervals (along with their appropriate interpretation) for judging study precision rather than focusing on statistical significance. Still, the fact that much of the published literature consists of studies in which the p-value is <0.05 (even if the authors do not focus on significance or even include p-values, NHST can be done by reviewers or editors using confidence intervals) suggests that change is moving slowly [11]. A call from the American Statistical Society and a follow-up editorial by Wasserstein et al. to move into a post *p* < 0.05 era [12,13] has sparked new interest in change, and a lengthy debate has begun on whether to move away from NHST entirely or to simply change the alpha used within the procedure has ensued [[14], [15], [16]]. Still, despite both appeals for change decades ago and renewed calls and energy for a move away from NHST, it is not clear the extent to which change has actually occurred.

A definitive way to answer this question is to review the published literature, either through what is reported in abstracts or through full text searches. While previously this was a laborious and painstaking effort, the development of rule-based text mining and machine learning has made this a much easier task. A 2017 review of the use of *p*-values, confidence intervals and NHST in the epidemiologic and medical literature from 1975 to 2014 set out to look for changes in uses of p-values using rule-based text mining by evaluating almost 90,000 abstracts in PubMed [17]. They found that reporting of only NHST has been the most common approach with a more marked shift in increasing reporting of confidence intervals in the epidemiologic journals than in the medical journals. However, this review focused on general medical and epidemiology journals. As such, this review could not tell whether there are differences in the ways that subfields of medicine and epidemiology have changed over time. A second review showed that in cardiovascular journals, the most common statistical inference reporting style was NHST, but with small (5–9 %) increases in use of only confidence intervals over time depending on the journal [18]. This review suggests that subfields of medicine and epidemiology may be experiencing very different practices with respect to statistical inference over time. With the emergence of the HIV/AIDS pandemic in the 1980s and the HIV/AIDS journals that accompanied it, the development of the reporting of statistical inference and effect measures can be examined from the beginning in a new field. As such we set out to update and implement the rule-based text mining and machine learning methodology from the general review to focus on the HIV/AIDS literature.

## Material and methods

### Search and inclusion citeria

To assess trends in reporting of statistical inference in the abstracts of the HIV/AIDS literature over time we conducted a systematic review of published abstracts. Our review included all PubMed entries that had an abstract in the 10 HIV/AIDS journals with the highest impact factor (IF) in 2022 according to Clarivate's Journal Citation Reports (JCR) in 2023 (https://jcr.clarivate.com/). The 10 journals (in parentheses the National Library abbreviation and IF) included were: *The Lancet HIV* (Lancet HIV, IF 16.1), *Journal of the International AIDS Society* (J Int AIDS Soc, IF 6.0), *AIDS Patient Care and STDS* (AIDS Patient Care STDS, IF 4.9), *Current HIV/AIDS Reports* (Current HIV/AIDS Rep, IF 4.6), *AIDS and Behavior* (AIDS Behav, IF 4.4), *Current Opinion in HIV and AIDS* (Curr Opin HIV AIDS, IF 4.1), *AIDS* (AIDS, IF 3.8), *Journal of Acquired Immune Deficiency Syndromes (*J Acquir Immune Defic Syndr, IF 3.6) (which we note has changed names over time), *HIV Medicine* (HIV Med, IF 3.0) and *AIDS Research and Therapy* (AIDS Res The, IF 2.2). The HIV/AIDS field has seen a large increase in the number of journals over the past decades and as such, some of these journals have longer histories than do others. Because for some journals there were few abstracts in the initial publication years, we excluded initial publication years with fewer than 10 PubMed entries from the reporting style trend analysis. The search was conducted on October 30, 2023 and included all abstract for these journals from January 1, 1987 to December 31, 2022 inclusive. We then downloaded all PubMed entries for these ten journals that met our search criteria for review by our text mining algorithm.

### Abstract review and text mining

To review the abstracts and extract key parameters, we used a previously developed and validated rule-based text mining algorithm to identify the presence of confidence intervals, numerical *p*-values (e.g. *p* = 0.03) or comparisons of p-values with thresholds (e.g., *p* < 0.01), as well as language describing statistical significance [[17], [18], [19], [20], [21]]. Using a second text mining algorithm, we identified the reporting of effect-measure estimates for dichotomous outcomes (e.g. death, progression, relapse, etc.) analyzed as either person-count or person-time data. Included effect measures were Risk Differences (RD), Rate Differences, Risk Ratios (RR), Incidence Rate Ratios (IRR), Hazard Ratios, Odds Ratios (OR), Number Needed to Treat (NNT) and Number Needed to Harm (NNH). The algorithm is unable to distinguish between the different varieties of ORs (prevalence or incidence ORs).

*P*-values are reported in many different forms and styles, which poses a challenge for automated extraction from text. To address this issue, an iterative approach was employed, beginning with the construction of a comprehensive initial Regular Expression (RegEx) designed to capture the diverse ways *p*-values are presented. This RegEx was then used as an annotation tool to train a BioBERT model [22], enabling it to learn the contextual representations of *p*-values within text. Predictions made by the transformer-based model [23] on unlabeled PubMed abstracts were compared with the initial RegEx results, leading to the identification of additional *p*-value reporting styles that were not previously accounted for. These newly discovered styles were subsequently incorporated into the RegEx.

The extracted *p*-values were categorized into three distinct types: (1) numerical values of p-values if p-values were reported with an equals sign (e.g. for *p* = 0.03 the extracted numerical value is 0.03), (2) threshold values (regardless whether the threshold is <, ≤, >, or ≥) for p-values if p-value thresholds were reported (e.g. for *p* < 0.01 the extracted thresfold value is 0.01). For threshold p-values, both the numeric threshold and the inequality direction (including whether the operator was inclusive or exclusive) was extracted.

### Validation of the algorithm

To check the validity of the algorithm, for each of the key factors the algorithm was meant to detect and categorize, we randomly drew 14 abstracts per period, 1987–1991, 1992–1996, 1997–2001, 2002–2006, 2007–2011, 2012–2016, 2017–2022 with each sample containing 7 abstracts where the algorithm said the factor was present and 7 where the algorithm said the factor was absent. We validated three aspects of the algorithm: 1) its ability to categorize the presence of absence of any statistical inference; 2) the presence or absence of specific measures of statistical inference (confidence intervals, numerical *p*-values, p-value thresholds, and significance language); and 3) the assessment of the presence of specific measures of association/effect (Hazard Ratio, Rate Ratio, Risk Ratio, Odds Ratio, Rate Difference, Risk Difference, Number Needed to Treat, Number Needed to Harm).

We validated the algorithm by comparing the results of the algorithm to human reviewed and adjudicated assessments. We then calculated the sensitivity and specificity of the algorithm to correctly identify each factor and estimated a pooled sensitivity and pooled specificity across these four factors (confidence intervals, numerical *p*-values, p-value thresholds, and significance language). In addition, the occurrence of significance terminology was rated regardless of its meaning (substantively or statistically) as the algorithm could not distinguish between the two.

To further validate the enhanced regular expression for identifying *p*-values, we applied it to a dataset of 1000 abstracts that were manually annotated by Chavalarias et al. [24]. We then compared the algorithms extraction results against the manually curated data. The improved RegEx successfully identified 754 out of the 756 p-values present in the abstracts. In addition, it detected four additional p-value mentions that were missed during the manual annotation process by Chavalarias et al. [24]. This validation served as an additional benchmark for the RegEx's precision and highlighted the success of the iterative refinement process used to develop it.

To validate the algorithm in terms of effect-measure estimates of interest, we drew time-stratified random samples of abstracts for which the algorithm predicted the presence of an effect-measure estimate of interest. We also drew a time-stratified random sample of abstracts where the algorithm predicted the absence of an effect-measure estimate of interest, but a keyword (for relative effect measures: “ratio”, for difference measures of effect: “difference”) was present. We then manually checked if the algorithm was correct and calculated sensitivity and specificity.

### Statistical methods

Based on the four characteristics (confidence intervals, numerical *p*-values, p-value thresholds, and significance language) per abstract, we categorized each abstract that contained any statistical inference using three dichotomous yes/no variables: 1) those reporting confidence intervals; 2) those reporting *p*-values, either as numerical values or as thresholds; and 3) those reporting any significance language. In addition, we categorized every abstract with any statistical inference hierarchically into one of four mutually exclusive categories with categories with lower numbers prioritized over those with higher ones: 1) those reporting confidence intervals; 2) those reporting numerical *p*-values without confidence intervals; 3) those reporting p-value thresholds without confidence intervals and numerical p-values; and 4) those reporting significance language numerical p-values, p-value thresholds and confidence intervals. The classification into the reporting style of statistical inference was made independent of the presentation of effect measures related to dichotomous outcomes.

We described the distribution of reporting styles by journal and calendar year and for the most recent period 2017–2022. We estimated time trends using weighted nonparametric local regression smoothing (LOESS) [25,26]. For the LOESS, we calculated 95 % confidence intervals using the score method [27] and derived inverse variance weights for additional LOESS weighting.

## Results

A total of 41,730 PubMed entries for the 10 included journals were identified in our initial search. Of these 10,065 (24 %) were not accompanied by an abstract and were excluded from the analysis, leaving a total of 31,665 abstracts for analysis. The three journals with the highest number of abstracts were *AIDS* (*N* = 9611), *J Acquir Immune Defic Syndr* (8508) and *AIDS Behav* (4642) which together accounted for 72 % of all abstracts (Suppl. Table 1). These are three of the longest running journals (with the first two having started in the late 1980s). Two journals, *Current Opinion HIV/AIDS* and *Current HIV/AIDS Rep* largely feature review articles and do not present many estimates of statistical inference. As such they are excluded from most analyses even though their descriptive data are still presented.

**Fig. 1:**
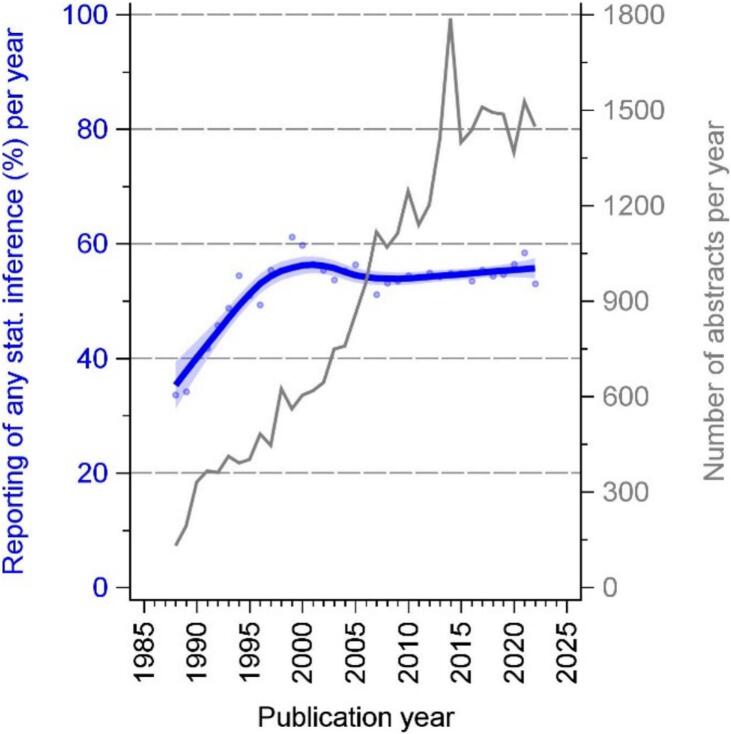
Number of abstracts per year and annual percentage of statistical inference reporting in 10 major HIV/AIDS journals from 1987 through 2022. **Legend: blue** indicates reporting of any statistical inference; **grey** indicates the number of abstracts per year. (For interpretation of the references to colour in this figure legend, the reader is referred to the web version of this article.)

**Fig. 2:**
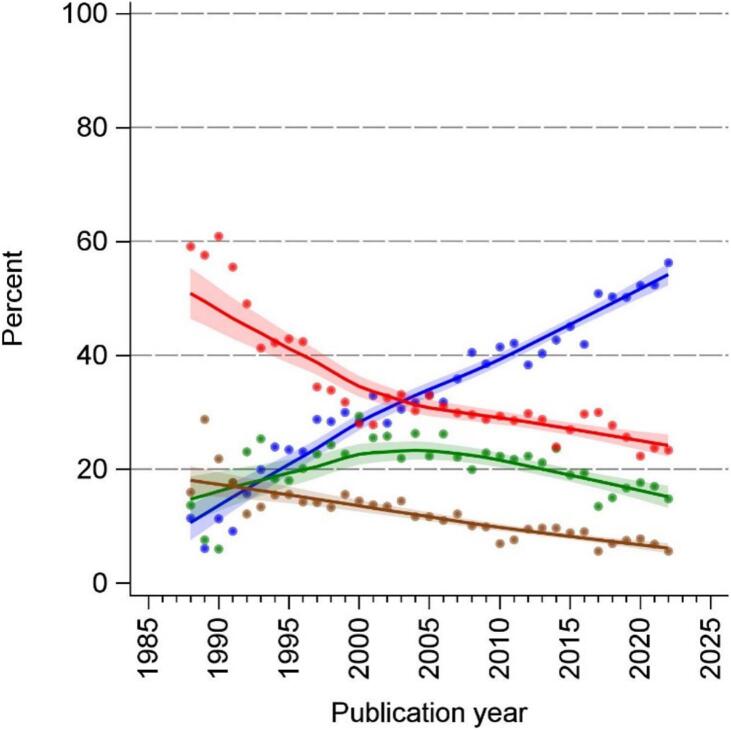
Annual reporting style on statistical inferences in abstracts that contain any statistical inference in 10 major HIV/AIDS journals from 1987 through 2022. **Legend**: **blue** indicates reporting of confidence intervals; **green** indicates reporting of numerical *p*-values (e.g. *p* = 0.03) without confidence intervals; **brown** indicates reporting of *p*-value thresholds (e.g. *p* < 0.01) without confidence intervals and numerical *p*-values; **red** indicates reporting of significance without confidence intervals, numerical p-values and *p*-value thresholds; all trend lines are LOESS smoothed with inverse-variance weighting; the colored bands present the 95 % confidence interval. (For interpretation of the references to colour in this figure legend, the reader is referred to the web version of this article.)

The annual number of abstracts in the 10 major HIV/AIDS journals increased from 131 (1988) to 1448 (2022). The proportion of abstracts containing statistical inference was 34 % in 1988. This proportion then rose to 61 % in 1999 and was roughly constant thereafter (Fig. 1). In the early years (1988, 59 %; 1996, 42 %), most abstracts reporting statistical inference contained only significance terminology without confidence intervals and *p*-values. This reporting style decreased throughout the observation period and especially in the early years 1988 to 2000. From 1988 to roughly 2005, each year about 30 % of all abstracts contained p-values without confidence intervals. Thereafter, this reporting style continued to decline. The reporting of confidence intervals increased steadily from 1988 (11 %) to 2022 (56 %) (Fig. 2).

The algorithm to identify the four characteristics related to statistical inference showed high validity with sensitivities ranging from 95 to 100 % and specificities ranging from 98 to 100 % for the four characteristics. The algorithm showed perfect sensitivity and specificity for the presence or absence of any report of statistical inference. Overall, this led to a pooled sensitivity of 98 % (95 %CI: 95–100) and pooled specificity of 100 % (95 %CI: 98–100). When it came to the measures of effect, while numbers were smaller, sensitivity was 98 % (95 %CI: 93–100) and specificity was 100 % (95 %CI: 99–100) (Suppl. Table 2).

Suppl. Fig. 1 presents the data by journal on the number of abstracts for each journal by year (in grey) and the prevalence of papers reporting of statistical inference in the abstracts (in blue) for the full time period of 1987–2022 along with LOESS smoothing trend lines. Excluding *Current Opin HIV/AIDS* and *Current HIV/AIDS Rep*, with the exception of *Lancet HIV,* which is a much newer journal with few abstracts in the early years, reports of any statistical inference have been increasing.

When we limited to 2017–2022, 8829 abstracts remained for analysis of reporting of statistical inference. Table 1 presents the data by journal on the prevalence of reporting of statistical inference in the abstracts and the different approaches to those reports. Of the 8829 abstracts (again excluding *Current Opin HIV/AIDS* and *Current HIV/AIDS Rep*), just over half (55.4 %) included any statistical inference, though it varied by journal, from 47.1 % in *AIDS Behav* to 66.8 % in *J Acquir Immune Defic Syndr*. Among the 4892 containing any reference to statistical inference, 52 % included a confidence interval whereas only 23.9 % include a confidence interval alone without *p*-values or assessments of significance. *The Lancet HIV* (89.1 %) and *J Acquir Immune Defic Syndr* (69.2 %) were the journals where abstracts were most likely to only include a confidence interval (*J Acquir Immune Defic Syndr* 43.5 % and *Lancet HIV* 35.6 %). Just under one third of abstracts contained numerical *p*-values while just under a quarter contained a p-value threshold. Interestingly, *Lancet HIV* was both most likely to include a confidence interval and most likely to include a numerical p-value. Just over half used some significance terminology.

**Table 1:** Prevalence of reporting of statistical inference in abstracts from 10 major HIV/AIDS journals, 2017–2022.

Journal	Total (n)	Any statistical inference (n)	Percent	Percentages among abstracts containing statistical inference (%)				
				Any CI	CI-only	Numerical p-value	p-value threshold	Significance terminology
**All journals**	**8829**	**4892**	**55.4**	**52.0**	**23.9**	**28.1**	**23.5**	**56.0**
AIDS	1624	1067	65.7	47.2	19.3	39.8	31.2	52.8
AIDS Behav	2199	1035	47.1	42.4	22.8	17.9	16.5	61.3
AIDS Patient Care STDS	348	177	50.9	43.5	13.0	31.1	33.3	65.0
AIDS Res Ther	362	224	61.9	54.0	16.1	32.1	34.4	68.8
*Curr HIV/AIDS Rep*	*288*	*50*	*17.4*	*4.0*	*2.0*	*2.0*	*0.0*	*98.0*
*Current Opin HIV/AIDS*	*348*	*68*	*19.5*	*4.4*	*1.5*	*1.5*	*0.0*	*98.5*
HIV Med	583	374	64.2	59.4	29.7	12.6	13.4	59.6
J Acquir Immune Defic Syndr	1718	1148	66.8	53.1	21.4	38.9	31.1	52.0
J Int AIDS Soc	952	510	53.6	69.2	43.5	6.7	6.3	51.6
Lancet HIV	407	239	58.7	89.1	35.6	46.0	30.1	31.0

CI – Confidence Intervals. Current HIV/AIDS Rep and Current Opin HIV/AIDS are not included in most analyses as they mainly publish review articles.

**Fig. 3:**
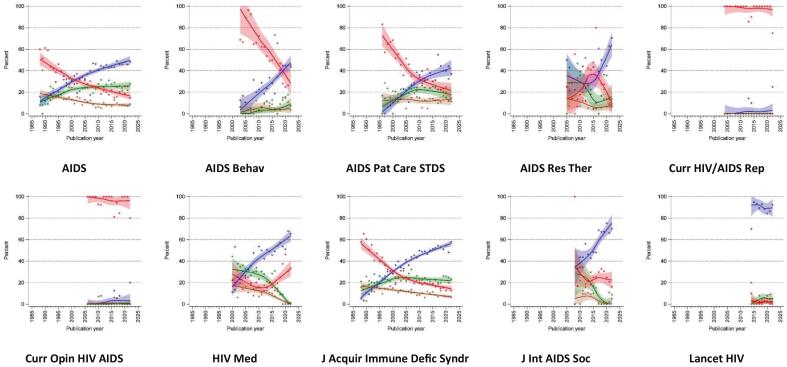
Flexibly estimated time trends of the statistical reporting style in abstracts of major HIV/AIDS journals (1987–2022) that contain any statistical inference. Legend: **blue** indicates reporting of confidence intervals; **green** indicates reporting of numerical p-values (e.g. *p* = 0.03) without confidence intervals; **brown** indicates reporting of p-value thresholds (e.g. *p* < 0.01) without confidence intervals and numerical p-values; **red** indicates reporting of significance without confidence intervals, numerical p-values and p-value thresholds; all trend lines are LOESS smoothed with inverse-variance weighting; the colored bands present the 95 % confidence interval. (For interpretation of the references to colour in this figure legend, the reader is referred to the web version of this article.)

Fig. 3 shows the way that reporting on statistical inference has changed over time by journal. Nearly all journals saw a decrease in reporting only significance without confidence intervals (red), and a rise in the percentage of studies reporting of confidence intervals (blue) with the exception of *Lancet HIV* which had nearly consistent high levels of reporting of only confidence intervals since its inception. No journal showed a strong increase in reporting on numerical *p*-values or p-value thresholds, but there were also few sharp declines with the exception of *J Acquir Immune Defic Syndr* and *HIV Med*.

Table 2 shows reporting of the prevalence of effect measure estimates from 2017 to 2022. Of the 16.6 % (1467/8829) of abstracts that included any effect measure, half reported odds ratios (50.5 %) suggesting a continued reliance on logistic regression, followed by hazard ratios (27.9 %) and risk ratios (15.7 %). Difference measures were very uncommon (2.6 % total with no journal reporting more than 4.6 %), as were number needed to treat (0.8 %) or to harm (0 %).

**Table 2:** Prevalence of reporting of various effect-measure estimates in abstracts of 10 major HIV/AIDS journals, 2017–2022.

Journal	Total (n)	Any effect-measure estimate n (%)		Percentages among abstracts containing effect-measure estimates (%)^a^							
				Hazard Ratio	Rate Ratio	Risk Ratio	Odds Ratio	Rate Diff.	Risk Diff.	NNT	NNH
**All journals**	**8829**	**1467**	**16.6**	**27.9**	**9.0**	**15.7**	**50.5**	**0.2**	**2.4**	**0.8**	**0**
AIDS	1624	351	21.6	32.8	12.3	15.4	43.3	0.3	2.9	1.1	0
AIDS Behav	2199	131	6.0	12.2	3.8	24.4	61.8	0	0.8	0	0
AIDS Patient Care STDS	348	66	19.0	12.1	6.1	9.1	74.2	0	1.5	0	0
AIDS Res Ther	362	43	11.9	20.9	2.3	16.3	62.8	0	0	2.3	0
*Curr HIV/AIDS Rep*	*288*	*0*									
*Current Opin HIV/AIDS*	*348*	*5*	*1.4*								
HIV Med	583	169	29.0	29.6	8.3	7.7	58.0	0	0	0.6	0
J Acquir Immune Defic Syndr	1718	430	25.0	29.8	7.2	14.0	50.0	0.2	2.8	0.9	0
J Int AIDS Soc	952	163	17.1	29.4	9.8	22.1	46.0	0	3.7	0.6	0
Lancet HIV	407	109	26.8	31.2	16.5	16.5	40.4	0.9	4.6	0.9	0

Legend: ^a^ the same abstract may have reported more than one type of effect-measure estimate so that the sum of percentages is above 100 %; Curr HIV/AIDS Rep and Current Opin HIV/AIDS are not included in most analyses as they mainly publish review articles. NNT – number needed to treat, NNH – number needed to harm, Diff – difference.

Suppl. Fig. 2 shows the distribution of *p*-values (p-curve) and p-thresholds across all journals for the period 1987–2022. There is a strong drop in the frequency of numerical p-values just above 0.05. The categorical p-value distribution over time within the 10 major HIV/AIDS journals and within the entire PubMed database (after exclusion of the 10 major HIV/AIDS journals) reveals that the time trends in HIV/AIDS journals are very similar with the entire PubMed database. The proportion of p-values below 0.05 decreased over time (Suppl. Fig. 3).

## Discussion

The debate over whether to move away from null hypothesis significance testing and replace it with estimation and precision using confidence intervals has gone on for decades and has received renewed attention recently with debates in the literature and a high profile more by the American Statistical Association noting a desire to move into the post *p* > 0.05 era. Our findings suggest that, within the HIV/AIDS literature, a field that only began in an era where there were prominent voices within epidemiology calling for change, there has been widespread use of confidence intervals. We found that most of the journals that we reviewed had a decrease in reporting only statistical significance without confidence intervals over time.

These results are similar to what was observed in the previous mentioned review of the published epidemiology and medical literature with a move away from only publishing null hypothesis significance testing approaches and towards more inclusion of confidence intervals. Still, it is encouraging to see that the HIV literature does appear to have more of a reliance on confidence intervals than the previously reviewed literature. We note that, while the move towards use of confidence intervals is a step in the right direction, if they are still only used to see whether the interval contains the null value, this is not as much progress as would be desired. Instead, calls for a focus on precision over statistical significance as called for by Poole [9] and others would be the preferred approach. Our methodology cannot distinguish between use of confidence intervals and the interpretation and future work should focus on seeing if the shift to more estimation and precision focused work is indeed occurring.

While the use of confidence intervals is encouraging, the observed continued focus on statistically significant results even as the move towards less significance testing was occurring, suggests that either reviewers and editors have been more focused on publishing results that were significant even if the authors did not use hypothesis testing, or that authors have been less likely to submit articles in which the results were not statistically significant. Another possible option is that most hypotheses being tested were indeed true positive results, but we consider this far less likely as the publication bias phenomenon is well established.

Our study findings should be considered in light of some limitations. First, our text mining algorithm and machine learning methodology, while showing high validity, was not perfect. As such, our findings suffer from some misclassification of the study results when they were categorized with respect to statistical inference reporting. While it is unlikely this would change the overall pattern of results we observed, there might be some change in the magnitude of the trend we observed. Second, we only looked at abstracts, which might not accurately represent the whole texts reporting style. For instance, based on 300 abstracts from the top three clinical pharmacology journals between 2012 and 2016, statistical inference was included in 50 % of the abstracts but 88 % of the complete texts of the same publications. Additionally, fewer abstracts with statistical inference included confidence intervals (45 %) than the complete texts of the same publications (58 %), which also included statistical inference [20]. Because (1) the abstract is frequently the only section of a publication that is read, (2) the reporting style in abstracts reflects the results that authors consider most noteworthy, and (3) proper presentation and interpretation of study results is relevant throughout the manuscript, it is especially relevant in abstracts [28], we decided to concentrate on abstracts. Third, our analyses did not distinguish whether the study was a randomized controlled trial or an observational study and it is possible that results depend on the study design.

## Conclusions

We see a marked improvement in the move away from reporting of only statistical significance language in the publications in the HIV/AIDS literature. While this alone does not signal a move away from hypothesis testing to the degree we would like to see, it does leave open the possibility that the move has happened. Even if it has not, it suggests fertile ground for moving towards a precision-based focus (focusing on the width of the confidence intervals, or how precisely we have measured what we say we have estimated) given that the transition to the use of confidence intervals has already largely happened.

## Patient approval statement

Not applicable.

## CRediT authorship contribution statement

**Andreas Stang:** Writing – review & editing, Writing – original draft, Visualization, Methodology, Investigation, Formal analysis, Conceptualization. **Henning Schäfer:** Writing – review & editing, Visualization, Formal analysis, Data curation. **Ahmad Idrissi-Yaghir:** Writing – review & editing, Methodology, Formal analysis, Data curation. **Christoph M. Friedrich:** Writing – review & editing, Supervision, Formal analysis, Conceptualization. **Matthew P. Fox:** Writing – review & editing, Writing – original draft, Supervision, Conceptualization.

## Ethics approval statement

Not applicable.

## Funding

The work of Henning Schäfer and Ahmad Idrissi-Yaghir was funded by a PhD grant from the 10.13039/501100001659Deutsche Forschungsgemeinschaft (DFG) Research Training Group 2535 “Knowledge- and data-based personalisation of medicine at the point of care” (WisPerMed).

## Declaration of competing interest

None of the authors declares any conflict of interest.

## Data Availability

Raw data are available at the web page of the journal.

## Appendix A Supplementary data

Supplementary material 1 Image 1

Supplementary material 2 Image 2

Supplementary material 3 Image 3

Supplementary material 4 Image 4

Supplementary material 5 Image 5

Supplementary material 6 Image 6

Supplementary material 7 Image 7

Supplementary material 8 Image 8
