# Supplementary material for: Statistical inference and effect measures in abstracts of major HIV and AIDS journals, 1987–2022: A systematic review
Source: Glob Epidemiol. 2025 Jul 25;10:100213. doi: 10.1016/j.gloepi.2025.100213 (PMC12337199; doi:10.1016/j.gloepi.2025.100213)
Supplement: Supplementary file 1 — Supplementary material 1 [file mmc1.docx]

**SUPPLEMENT: Stang et al. Statistical inference and effect measures in abstracts of major HIV and AIDS journals, 1987-2022: A systematic review**

**Suppl. Table 1**

**Available 31,665 abstracts in 10 major HIV/AIDS journals of the period 1987-2022**

| Pub year | AIDS | AIDS Behav | AIDS Pat Care STDS | AIDS Res Ther | Curr HIV/AIDS Rep | Current Opin HIV AIDS | HIV Med | J Acquir Immune Defic Snydr | J Int AIDS Soc | Lancet HIV | Total |
| --- | --- | --- | --- | --- | --- | --- | --- | --- | --- | --- | --- |
| 1987 | 34 |  |  |  |  |  |  |  |  |  | 34 |
| 1988 | 75 |  |  |  |  |  |  | 56 |  |  | 131 |
| 1989 | 118 |  |  |  |  |  |  | 75 |  |  | 193 |
| 1990 | 168 |  |  |  |  |  |  | 163 |  |  | 331 |
| 1991 | 211 |  |  |  |  |  |  | 155 |  |  | 366 |
| 1992 | 183 |  |  |  |  |  |  | 178 |  |  | 361 |
| 1993 | 227 |  |  |  |  |  |  | 185 |  |  | 412 |
| 1994 | 221 |  |  |  |  |  |  | 170 |  |  | 391 |
| 1995 | 178 |  |  |  |  |  |  | 224 |  |  | 402 |
| 1996 | 236 |  | 26 |  |  |  |  | 220 |  |  | 482 |
| 1997 | 236 | 1 | 17 |  |  |  |  | 192 |  |  | 446 |
| 1998 | 317 | 0 | 75 |  |  |  |  | 232 |  |  | 624 |
| 1999 | 291 | 1 | 60 |  |  |  | 7 | 202 |  |  | 561 |
| 2000 | 332 | 1 | 61 |  |  |  | 18 | 192 |  |  | 604 |
| 2001 | 310 | 1 | 68 |  |  |  | 32 | 207 |  |  | 618 |
| 2002 | 305 | 1 | 64 |  |  |  | 33 | 241 |  |  | 644 |
| 2003 | 340 | 39 | 59 |  |  |  | 39 | 272 |  |  | 749 |
| 2004 | 323 | 43 | 65 | 2 | 25 |  | 61 | 239 |  |  | 758 |
| 2005 | 338 | 55 | 87 | 11 | 29 |  | 61 | 267 | 9 |  | 857 |
| 2006 | 299 | 87 | 82 | 27 | 27 | 68 | 75 | 291 | 1 |  | 957 |
| 2007 | 424 | 118 | 105 | 28 | 28 | 66 | 79 | 267 | 2 |  | 1117 |
| 2008 | 343 | 117 | 98 | 26 | 26 | 92 | 98 | 257 | 12 |  | 1069 |
| 2009 | 306 | 140 | 125 | 30 | 27 | 76 | 83 | 281 | 44 |  | 1112 |
| 2010 | 382 | 169 | 89 | 47 | 30 | 73 | 80 | 309 | 66 |  | 1245 |
| 2011 | 260 | 208 | 88 | 44 | 32 | 81 | 72 | 280 | 74 |  | 1139 |
| 2012 | 281 | 250 | 84 | 38 | 40 | 75 | 76 | 289 | 69 |  | 1202 |
| 2013 | 338 | 327 | 78 | 31 | 45 | 72 | 93 | 322 | 103 |  | 1409 |
| 2014 | 335 | 308 | 78 | 39 | 50 | 75 | 75 | 361 | ^ⴕ^455 | 12 | 1788 |
| 2015 | 287 | 251 | 81 | 43 | 56 | 67 | 94 | 348 | 125 | 46 | 1398 |
| 2016 | 289 | 326 | 66 | 41 | 45 | 84 | 81 | 315 | 140 | 49 | 1436 |
| 2017 | 292 | 358 | 59 | 60 | 23 | 81 | 86 | 341 | 159 | 50 | 1509 |
| 2018 | 303 | 399 | 59 | 28 | 46 | 71 | 101 | 271 | 144 | 70 | 1492 |
| 2019 | 275 | 358 | 55 | 40 | 53 | 67 | 84 | 310 | 179 | 66 | 1487 |
| 2020 | 249 | 312 | 53 | 67 | 61 | 47 | 80 | 258 | 168 | 72 | 1367 |
| 2021 | 269 | 404 | 58 | 99 | 49 | 38 | 104 | 272 | 162 | 71 | 1526 |
| 2022 | 236 | 368 | 64 | 68 | 56 | 44 | 128 | 266 | 140 | 78 | 1448 |
|  |  |  |  |  |  |  |  |  |  |  |  |
| Total | 9611 | 4642 | 1904 | 769 | 748 | 1177 | 1740 | 8508 | 2052 | 514 | 31665 |

Legend eTable 1: blue figures: starting year for studying the style of reporting of statistical inference (at least n=10 abstracts within a year available); ^ⴕ^ increased due to publication of conference proceeding for HIV Drug Therapy in the Americas. Pub – publication.

**Suppl. Table 2**

**Estimated sensitivity and specificity of the algorithm to identify the four characteristics related to statistical inference and to identify measures of association**

| **Reporting style** | **Sensitivity** | **%** | **Specificity** | **%** |
| --- | --- | --- | --- | --- |
| Any statistical inference | 47/47 | 100 | 51/51 | 100 |
|  |  |  |  |  |
| Characteristics related to statistical inference |  |  |  |  |
| Confidence interval reporting | 22/23 | 95 | 75/75 | 100 |
| Numerical p-value reporting | 32/33 | 97 | 64/65 | 98 |
| P-value threshold reporting | 32/33 | 97 | 63/63 | 100 |
| Significance terminology | 72/72 | 100 | 26/26 | 100 |
|  |  |  |  |  |
| Across all four characteristics related to statistical inference | 158/161 | 98 | 228/229 | 100 |
|  |  |  |  |  |
| Measures of effect |  |  |  |  |
| Hazard ratio | 13/14 | 93 | 84/84 | 100 |
| Odds ratio | 59/59 | 100 | 38/39 | 97 |
| Rate difference | 1/1 |  | 97/97 | 100 |
| Rate ratio | 6/6 |  | 92/92 | 100 |
| Risk ratio | 20/21 | 95 | 77/77 | 100 |
|  |  |  |  |  |
| Across all measures of association | 99/101 | 98 | 388/389 | 100 |

Legend: The algorithm and the rater neither found the following measures of association in the validation sample: risk difference, number needed to treat, number needed to harm; note – the occurrence of significance terminology was rated regardless of its meaning (substantively or statistically).

**Suppl. Figure 1 Annual number of abstracts and annual percentage of abstracts containing statistical inference in 10 major HIV/AIDS journals, 1987-2022**

| 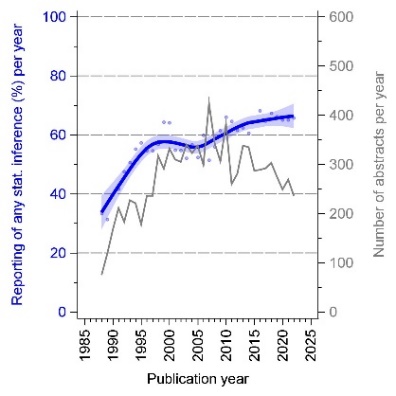 | 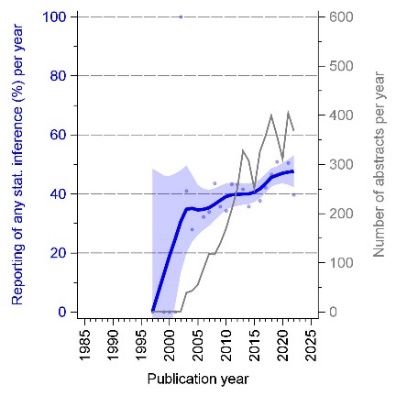 | 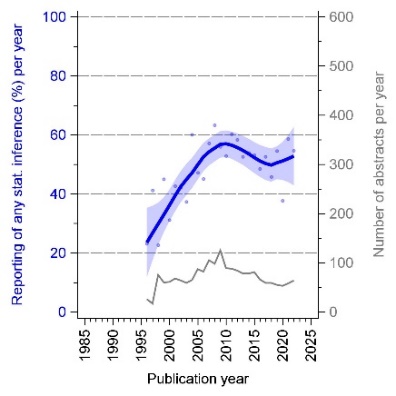 | 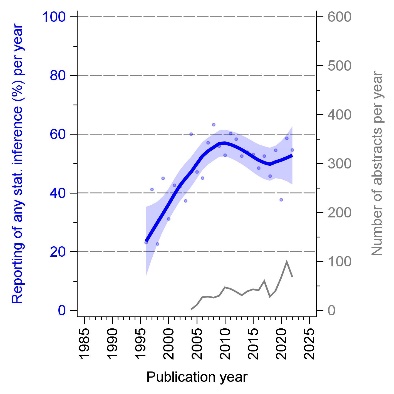 | 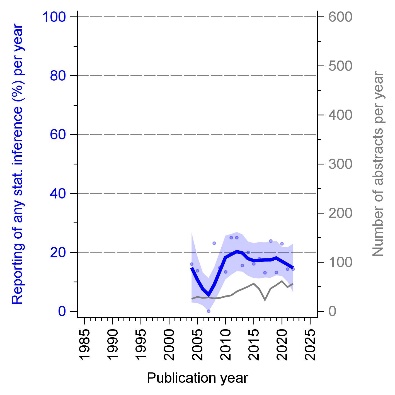 |
| --- | --- | --- | --- | --- |
| **AIDS** | **AIDS Behav** | **AIDS Pat Care STDS** | **AIDS Res Ther** | **Curr HIV/AIDS Rep** |
| 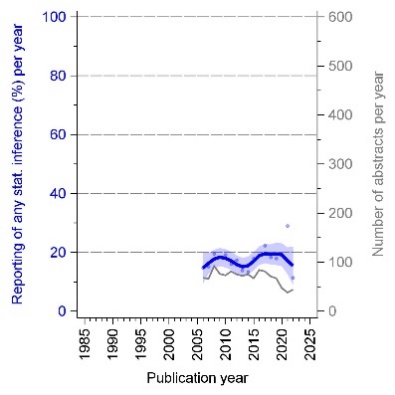 | 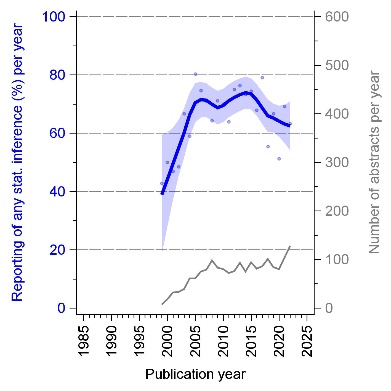 | 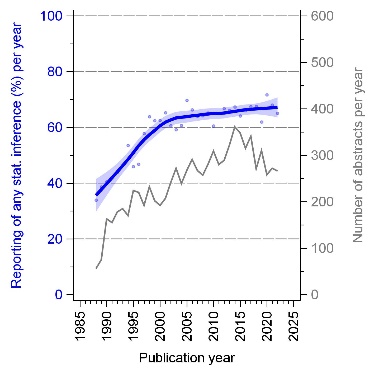 | 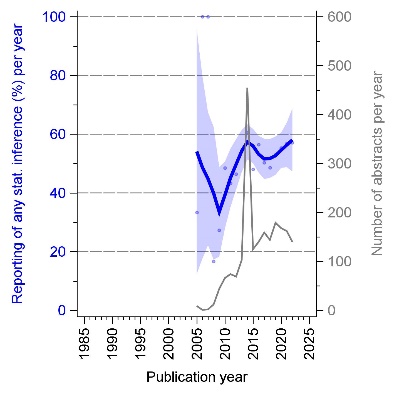 | 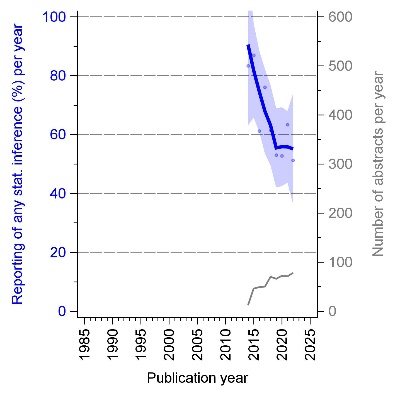 |
| **Curr Opin HIV AIDS** | **HIV Med** | **J Acquir Immune Defic Syndr** | **J Int AIDS Soc** | **Lancet HIV** |

Legend: Grey line represents the number of publications (left Y axis), blue line represents the proportion of abstracts reporting any statistical significance (right Y axis). Trend lines for the percentage of statistical inference are LOESS smoothed with inverse-variance weighting; the colored bands present the 95% confidence interval.

**Suppl. Figure 2**

**Distribution of 14,761 numerical p-values (blue) and 9,635 p-value thresholds (red) in abstracts of 10 major HIV/AIDS journals of the years 1987-2022**


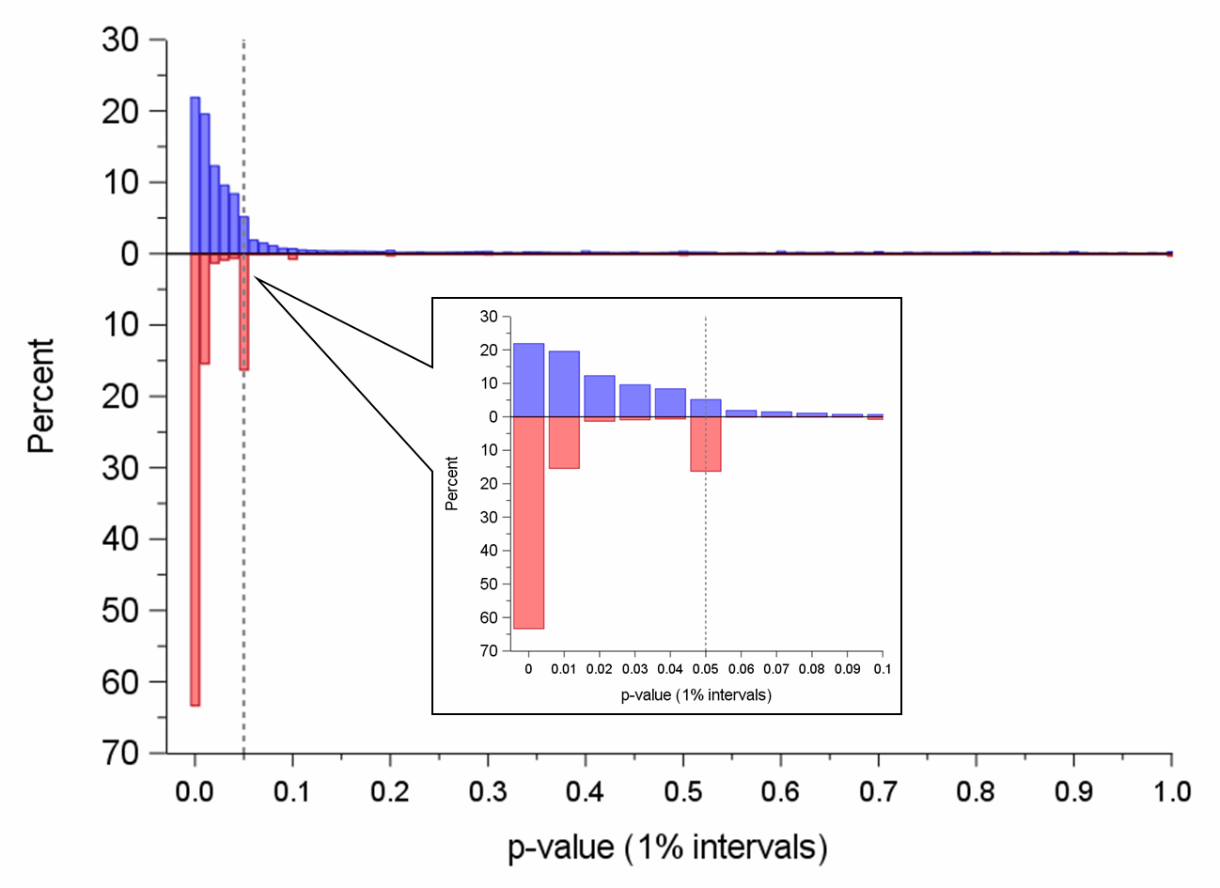


All numerical p-values (n=14,761) were rounded to 2 decimal places; P-value thresholds (p<, p≤, p>, p≥, n=9,635) were numerically extracted and rounded to 2 decimal places; P-values coded as zero contain p-values < 0.005 (i.e., p=0.0049 or less, rounded to 2 decimal places, therefore zero); Blue bars illustrate the distribution of numerical p-values, red bars show the distribution of p-value thresholds;

**Suppl. Figure 3 Time trends in the distribution of numerical p-value categories in 10 major HIV/AIDS journal abstracts compared to the entire PubMed database (1987–2022)**

| **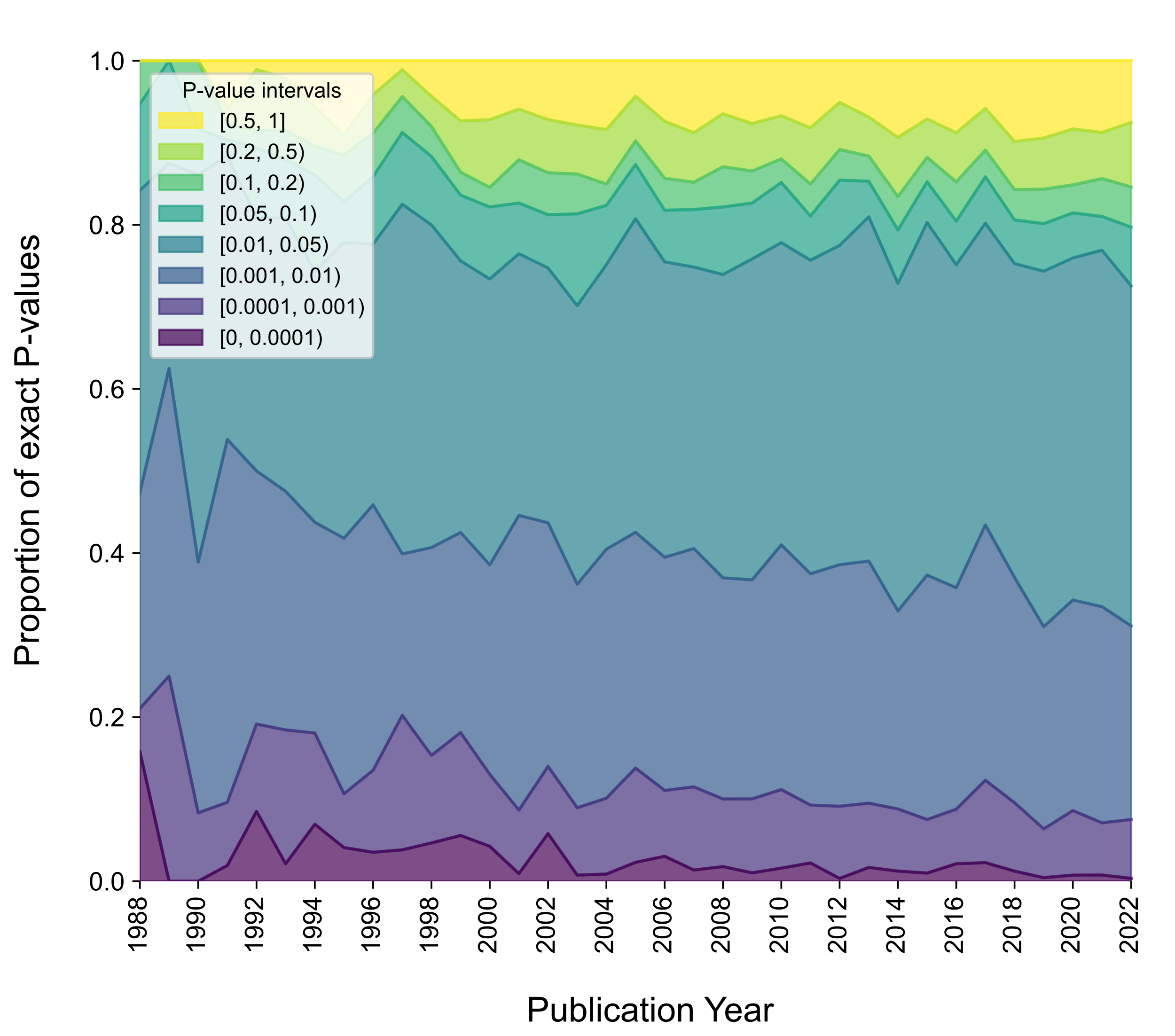** | **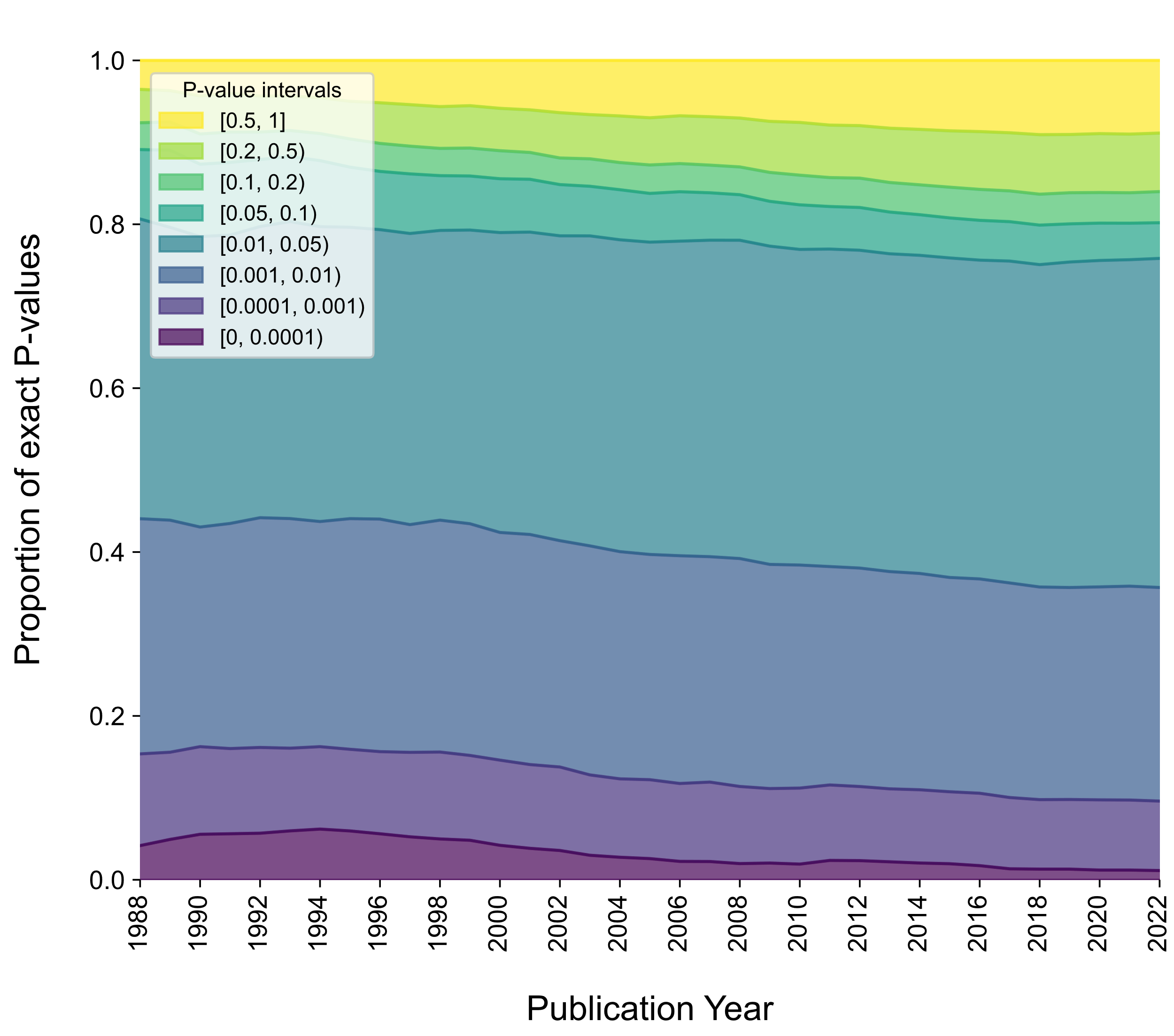** |
| --- | --- |
| **HIV/AIDS** | **Entire PubMed database** |

The plots show the proportion of reported p-values by p-value categories over time for a direct comparison of trends in statistical reporting practices. HIV/AIDS-specific journals were excluded from the overall PubMed dataset to avoid any overlap.
