# Supplementary material for: Statistical inference and effect measures in abstracts of major HIV and AIDS journals, 1987–2022: A systematic review
Source: Glob Epidemiol. 2025 Jul 25;10:100213. doi: 10.1016/j.gloepi.2025.100213 (PMC12337199; doi:10.1016/j.gloepi.2025.100213)
Supplement: Supplementary file 2 — Supplementary material 2 [file mmc2.docx]

**Supplementary File: Metadata**

**Raw data file for Table 1, Table 2, Figure 1, Figure 2, Figure 3, Suppl. Table 1, Suppl. Figure 1**

| **Excel file name** | **Variable name** | **Coding** |
| --- | --- | --- |
| **MainDataFile.zip**  **(n=43417 observations)**  **[zipped excel file]** | PMID  (PubMed ID) | ID |
|  | TA  (Journal name) | AIDS  AIDS Behav  AIDS Patient Care STDS  AIDS Res Ther  Curr HIV/AIDS Rep  Curr Opin HIV AIDS  HIV Med  J Acquir Immune Defic Syndr  J Acquir Immune Defic Syndr (1988)  J Acquir Immune Defic Syndr Hum Retrovirol  J Int AIDS Soc  Lancet HIV |
|  | Pubyear | 1987-2022 |
|  | Empty* | 1: no abstract  0: abstract present |
|  | P_use | Presence of p-values (numeric andor threshold) |
|  | CIany | Presence of confidence intevals |
|  | SigTerm | Presence of “significance terminology” |
|  | NNT | 0: absent, 1: present |
|  | NNH | 0: absent, 1: present |
|  | RateDiff | 0: absent, 1: present |
|  | RiskDiff1 | 0: absent, 1: present |
|  | RateRatio | 0: absent, 1: present |
|  | RiskRatio | 0: absent, 1: present |
|  | HazardRatio | 0: absent, 1: present |
|  | OddsRatio1 | 0: absent, 1: present |

*n=10065 out of 41730 PubMed entrances have no abstract

**Raw data related to Suppl. Figure 2**

| **Excel file name** | **Content** | **Variable name** | **Coding** |
| --- | --- | --- | --- |
| **LowerP.xls**  **(n=9360 observations)** | Contains all p-values reported as threshold:  P < … or P ≤ … | PMID  (PubMed ID) | ID |
|  |  | Inclusive | 0: P <  1: P ≤ |
|  |  | Lower_p_value | Numerical threshold value |
|  |  |  |  |
| **GreaterP.xls**  **(n=275 observations)** | Contains all p-values reported as threshold:  P > … or P ≥ … | PMID  (PubMed ID) | ID |
|  |  | Inclusive | 0: P <  1: P ≤ |
|  |  | Greater_p_value | Numerical threshold value |
|  |  |  |  |
| **ExactP.xls**  **(n=14761 observations)** | Contains all exactly reported p-values | PMID  (PubMed ID) | ID |
|  |  | Pvalue | Numerical p-value |

**Raw data related to Suppl. Figure 3**

| **ZIP file name** | **Content** | **Variable name** | **Coding** |
| --- | --- | --- | --- |
| **Hiv_p_values_all_years.zip**  **(n=14814 observations)** | csv data set containing all extracted p-values among 10 major HIV/AIDS journals 1987-2022 | PMID  (PubMed ID) | ID |
|  |  | Exact_p_value | Numerical p-value |
|  |  | Publication_year | 1987-2022 |
|  |  |  |  |
| **PubMed_p_values_all_years.zip**  **(n=4.231.552 observations)** | csv data set containing all extracted p-values in Pubmed 1987-2022 (without 10 major HIV/AIDS journals 1987-2022) | PMID  (PubMed ID) | ID |
|  |  | Exact_p_value | Numerical p-value |
|  |  | Publication_year | 1987-2022 |
